# Depicting Soybean Diversity via Complementary Application of Three Marker Types

**DOI:** 10.3390/plants14020201

**Published:** 2025-01-12

**Authors:** Vesna Perić, Natalija Kravić, Marijenka Tabaković, Snežana Mladenović Drinić, Valentina Nikolić, Marijana Simić, Ana Nikolić

**Affiliations:** Maize Research Institute Zemun Polje, Slobodana Bajića 1, 11185 Belgrade, Serbia; nkravic@mrizp.rs (N.K.); mtabakovic@mrizp.rs (M.T.); drinicsnezana2@gmail.com (S.M.D.); valentinas@mrizp.rs (V.N.); marijana.simic@mrizp.rs (M.S.); anikolic@mrizp.rs (A.N.)

**Keywords:** agronomic traits, genetic resources, *Glycine max* (L.) Merr., HOMALS, morphological descriptors, multivariate methods, SSR, working collection

## Abstract

Driven by the growing demands for plant-based protein in Europe and attempts of soybean breeding programs to improve the productivity of created varieties, this study aimed to enhance genetic resource utilization efficiency by providing information relevant to well-focused breeding targets. A set of 90 accessions was subjected to a comprehensive assessment of genetic diversity in a soybean working collection using three marker types: morphological descriptors, agronomic traits, and SSRs. Genotype grouping patterns varied among the markers, displaying the best congruence with pedigree data and maturity for SSRs and agronomic traits, respectively. The clear origin-related grouping pattern was not observed for any of the marker types. For the diversity assessed by morphological descriptors, Homogeneity Analysis by Means of Alternating Least Squares (HOMALS) yielded the most efficient classification by identifying the traits with the highest discriminative power and separating the genotypes into homogeneous groups. According to genetic distances (GDs), the highest diversity was found for morphological descriptors (GD = 517), followed by SSRs (GD = 0.317) and agronomic traits (GD = 0.244). The analysis of molecular variance (AMOVA) revealed a weak differentiation between geographic groups (Φ*_ST_* = 0.061), emphasizing the highest differentiation for Canadian genotypes (Φ*_ST_* = 0.148 **). A low correlation was found between molecular and morphological, i.e., agronomic trait-based matrices (0.061 *, i.e., –0.027, respectively). The overall assessed diversity highlighted the importance of introducing new sources of variation to promote long-term improvement in soybean breeding.

## 1. Introduction

The importance of soybean in the global food chain stems from the quality of the grain, which contains 17–22% oil and 35–47% protein rich in important amino acids, placing this plant species in a strategic position in the world’s production of oil and plant-based proteins [1]. With the development of the processing industry and the appearance of alternative ways of soybean utilization, the soybean growing area in Serbia is constantly expanding, reaching 235,275 ha in 2022, with an average yield of 2.42 t/ha. With a production of 398,556 tons per year, Serbia ranks second in Europe, right after the Russian Federation [2]. The expansion of soybean acreage in Serbia aligns with the European trend of increasing soybean production, aiming to provide domestic sources of plant-based protein and minimize dependency on overseas imports [3]. Although soybean breeding programs worldwide display a wide range of breeding objectives, selection for seed yield and maturity are still the most important breeding goals [4]. Over the last 60 years, the average global soybean yield increased from 1.13 t/ha in 1960 to 2.77 t/ha in 2020 [5]. This progress is mostly attributed to genetic improvement in seed yield and traits determining tolerance/resistance to abiotic and biotic stress [4,6].

Soybean breeders face numerous challenges in their efforts to develop high-yielding varieties with favourable seed composition and stable agronomic performance, including a lack of genetic diversity, limited soybean adaptation to regional photo-thermal conditions, and unprecedented climate changes. Soybean has undergone several processes of genetic diversity erosion: early domestication, founding events, and intensive modern breeding [7,8]. Breeding efforts have led to an increase in the frequency of favourable alleles, resulting in their fixation and thereby reducing genetic diversity [9,10].

The narrowing of the soybean genetic base is reinforced by the breeders’ tendency to use biparental crosses of elite lines for the development of segregating generations [11], as well as the establishment of a plant variety protection system that restricts material exchange between breeding companies [12]. In addition to breeding challenges, the adaptive response of soybean cultivars introduced to new regions is often hard to predict due to the complexity of the photoperiod, temperature sum, and water availability [13,14]. The photoperiodic response of soybean is conditioned by the allele distribution at loci determining flowering and maturity and largely determines cultivars’ geographical distribution and latitudinal adaptation [15,16]. Finally, climate changes put new challenges ahead of breeders, requiring modification of the breeding objectives and a fast response to growing food demands [17].

To ensure continuous progress in breeding, future strategies should focus on the identification and provision of novel sources of variation within the global genetic resources. A systematic and accurate phenotypic evaluation of the germplasm in the collections is a prerequisite for its effective utilization in breeding [18]. Agro-morphological characterization enables assessing genetic diversity, creating the core collections, classifying materials from various geographic regions, and identifying the priority accessions for the best possible use in breeding programs [19,20,21,22]. Generally, the evaluation involves recording important agronomic traits and morphological descriptors, i.e., phenotypic markers. Quantitative and qualitative data sets are employed for inferring genetic distances [23], assuming that phenotypic differences between cultivars approximate their genetic diversity [19]. Numerous authors stated that phenotypic markers were unreliable or, at best, only indirect indicators of genetic diversity due to their limited number, low polymorphism, expression influenced by environmental factors, high selection pressure, observers’ subjectivity, and limitations of the established evaluation systems [24,25,26]. However, from the perspective of breeders, diversity estimates based on phenotypic data, while accounting for just a small percentage of overall diversity, remain the most relevant information for well-focused breeding applications [27]. In addition, a large number of accessions evaluated for multiple traits in multi-location trials enhance the accuracy of genetic diversity predictions on the basis of phenotypic variation [19,28]. In contrast to the phenotypic markers, the high polymorphism of microsatellite (simple sequence repeats, SSRs) markers, their abundance and wide coverage of the genome, their multi-allelic nature, and the high reproducibility of the results make SSRs an irreplaceable tool for quantifying genetic diversity in the population, discovering the patterns of differentiation, and mapping the loci of interest [29,30,31].

The germplasm studied in this research is a part of the soybean working collection maintained at the Maize Research Institute Zemun Polje (MRIZP), Serbia. The collection dates back to the early eighties and comprises approximately 400 introduced accessions from regions worldwide and 200 released indigenous former Yugoslavian varieties. Although the resources from the collection are intensively used for different breeding-orientated purposes, both the accurate and systematic evaluation and the assessment of the stored material diversity have not yet been carried out. Information on accessions comprises passport data, incomplete pedigree information, and poor morphological description. Hence, an improved understanding of local small working collections with adapted germplasm that had already been developed during pre-breeding efforts highlighted their significant applicability, making them an excellent source for a variety of breeding objectives. These findings prompted us to conduct a comprehensive characterization of the accessions in the collection by evaluating a representative set of 90 soybean genotypes using three marker types: agronomic traits, morphological descriptors, and SSR markers. The objectives of this study were to (i) quantify the level of diversity in the collection, (ii) identify underlying patterns of phenotypic and molecular differentiation among studied genotypes, and (iii) estimate a potential of phenotypic-derived genetic distances for relevant diversity assessment at the molecular level.

## 2. Results

### 2.1. Differentiation Pattern Revealed by Morphological Traits

#### 2.1.1. HOMALS Analysis

**Figure 1:**
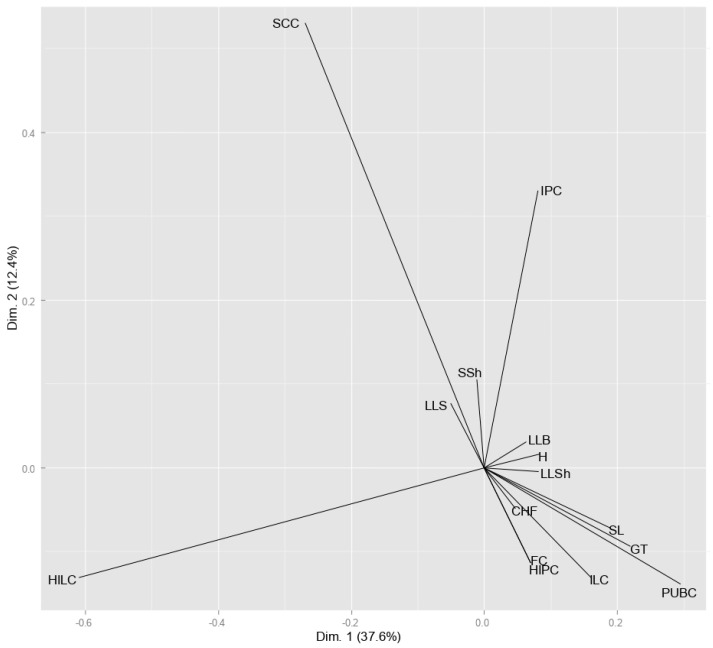
Discriminative power of 15 morphological descriptors according to HOMALS analysis. Abbreviations: HIPC—hypocotyl colour; H—habitus; GT—growth type; PUBC—pubescence colour; LB—leaf blistering; LLSh—shape of lateral leaflet; LLS—size of lateral leaflet; ILC—intensity of the green colour of the leaf; FC—flower colour; IPC—intensity of the brown colour of the pod; SSh–seed shape; SCC—seed coat colour; SL—seed coat lustre; HILC—hilum colour; CHF—colour of hilum funicle.

**Figure 2:**
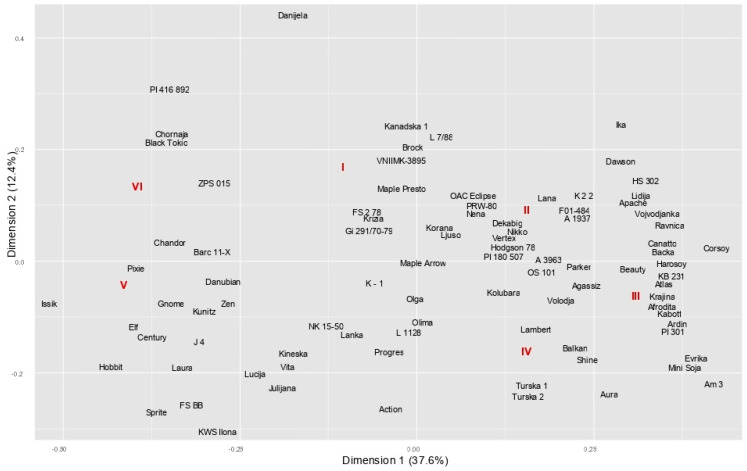
Grouping of genotypes derived by HOMALS analysis based on 15 morphological descriptors. Roman numbers in red represent groups of genotypes with a similar morphological profile.

Visual scores for fifteen morphological descriptors in 90 soybean genotypes (Table S1) were subjected to HOMALS to reveal the discriminative power of the descriptors and determine the grouping pattern among the genotypes. The first axis explained the largest part of the total variation (37.6%), while the second axis accounted for 12.4% of the variability contained in the original data set (Figure 1 and Figure 2). The studied morphological descriptors expressed different magnitudes of variation as indicated by vector length, determining the genotypes’ division along two dimensions of the scatterplot. The first dimension was mainly related to the descriptors HILC—hilum colour and PUBC—pubescence colour, and to a lesser extent to GT—growth type and ILC—intensity of the green colour of the leaf. Dimension 2 was associated with the descriptors IPC—intensity of the brown colour of the pod and SCC—seed coat colour, which had high loadings on the second axis. The longest vectors were observed for descriptors HILC, SCC, PUBC, and IPC, suggesting a high discriminative power of these traits. Short vectors of descriptors located close to the origin (LLS—size of lateral leaflet, SSh—seed shape, LB—leaf blistering, H—habitus, LLSh—shape of lateral leaflet, CHF—colour of hilum funicle, FC—flower colour, SL—seed coat lustre, and HIPC—hypocotyl colour) indicated their poor discriminative power.

A considerable variation in the studied descriptors across the genotypes led to their broad dispersion on the scatterplot (Figure 2). Genotypes with similar morphological profiles were classified into homogeneous and moderately separated groups: group I—genotypes of yellow pubescence and brown or dark brown hilum; group II—genotypes of yellow or light-brown hilum, yellow seed coat, dark pods, and indeterminate growth; group III—genotypes of grey pubescence, yellow hilum, yellow seed coat, and indeterminate growth type; group IV—genotypes of grey pubescence, light-brown hilum, indeterminate growth type, and yellow seed coat; group V—genotypes of yellow pubescence, black hilum, and determinate or indeterminate growth type; and group VI—genotypes with coloured seed coat (black, brown, olive).

Genotypes with distinct profiles were positioned farther from the coordinate origin. The existence of certain categories of descriptors unique to a particular genotype was discovered in the cultivar Danijela (brown seed coat) and exotic germplasm Barc 11-X (fasciata growth type). The plot of object scores revealed no genotypes with identical object scores, i.e., identical profiles. With the majority of its attributes matching the most frequent category of descriptors, the cultivar Maple Arrow was positioned in the coordinate origin and considered a “standard profile”.

The HOMALS-derived grouping demonstrated relatively poor agreement with pedigree data and information on geographic origin (Table S2). The highest consistency in the grouping with respect to pedigree was determined for group V, which encompassed seven genotypes traced back to the American variety Williams. Six of the ten Canadian genotypes clustered in group I, including the varieties Maple Arrow, Maple Presto, and OAC Eclipse, shared the common parentage. Group III encompassed five of the seven genotypes developed by the same breeding institution, of which three cultivars (Vojvođanka, Ravnica, and Afrodita) represent progeny from the same cross and were closely located to their progenitors—the old varieties Hodgson 78, Corsoy, and Harosoy. The three latter genotypes were in close proximity to the American genotypes Dawson, Lambert, Parker, and A 1937, the Romanian variety F01-484, and the domestic variety Balkan, all of which were pedigree-related.

#### 2.1.2. Cluster Analysis Based on Morphological Descriptors

Genetic distances calculated on the basis of the similarity in morphological profiles in the 90 soybean genotypes are presented in Table S3. The smallest genetic distance (GD = 0.063) was determined for three pairs of genotypes, i.e., Apache–Harosoy, Bačka–Kolubara, and Harosoy–KB 231, while the largest genetic distance (GD = 1.000) was observed for the Canatto–L 7/88 and Shine–PI 416 892 pairs. The examined genotypes exhibited a relatively high level of morphological diversity, as indicated by the average genetic distance (GD = 0.517).

**Figure 3:**
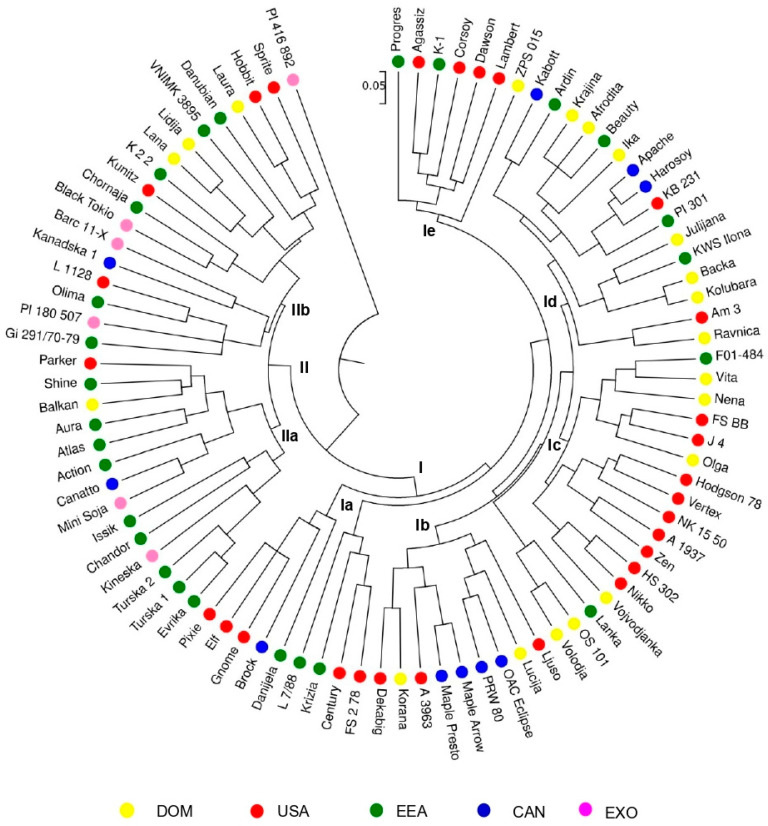
Dendrogram of UPGMA cluster analysis of 90 soybean genotypes based on 15 morphological descriptors. Genotypes labelled by geographic origin: DOM—domestic; Serbia and Croatia; USA—United States of America; EEA—Europe and Euro-Asia; CAN—Canada; EXO—exotic; China and Japan.

The UPGMA (unweighted pair group method with arithmetic mean) dendrogram based on morphological descriptors clearly divided soybean genotypes into two main clusters, corresponding to flower and hypocotyl colour (I—purple; II—white) (Figure 3). All genotypes of white flowers (II) were further divided according to the pubescence colour into groups of grey (IIa) and yellow pubescence (IIb). Within cluster I, genotypes were further classified into five subclusters—Ia, Ib, Ic, Id, and Ie. Subclusters Ic, Id, and Ie consisted exclusively of genotypes of the indeterminate growth type; the remaining subclusters did not exhibit clear growth-type grouping. The highest genetic distance was observed for PI 416-892 due to its unique morphological profile.

Similar to the HOMALS analysis, in the grouping pattern obtained by cluster analysis, no dominant geographical component of morphological variability could be observed. The majority of the genotypes were not consistently grouped into subclusters with respect to the region of origin. The exception was observed for the nine closely clustered American varieties in subcluster Ic and individual pairs of genotypes of the same geographic origin and/or same breeding program (genotypes Turska 1 and Turska 2 in IIa; the Canadian varieties OAC Eclipse, Maple Arrow, Maple Presto, and PRW 80 in Ib; and cultivars developed in the same breeding program—Krajina, Afrodita, Bačka, Kolubara, and Ravnica in Id).

The pedigree-related grouping could only be observed for individual pairs of genotypes or a few weakly consistent groups within subclusters. Subcluster Ia contained the semi-dwarf genotypes Gnome, Elf, and Pixie, developed from the same cross. Three Canadian varieties (OAC Eclipse, Maple Arrow, and Maple Presto), derived from the Swedish genotype Holmberg 840-7-3, clustered together (subcluster Ib). Indirect descendants of the American cultivar Hodgson 78 (Afrodita, Ravnica, Vojvođanka, F01-484 and A 1937) were allocated to the same subcluster (Ic). A close grouping of the Canadian variety Apache to its progenitor Harosoy was observed in subcluster Id. Subcluster Ie gathered the cultivars Agassiz, Dawson, Lambert, and Corsoy, all derived from the old Canadian variety Harosoy. The cultivars Canatto, Balkan, and Parker, which share a different percentage of the germplasm of the American variety Evans, were assigned to the same group in subcluster IIa. A Williams-based group within subcluster IIb encompassed the cultivars Lana, Lidija, Laura, Kunitz, Hobbit, and Sprite.

### 2.2. Diversity Pattern Revealed by Agronomic Traits

#### 2.2.1. PC Analysis

**Figure 4:**
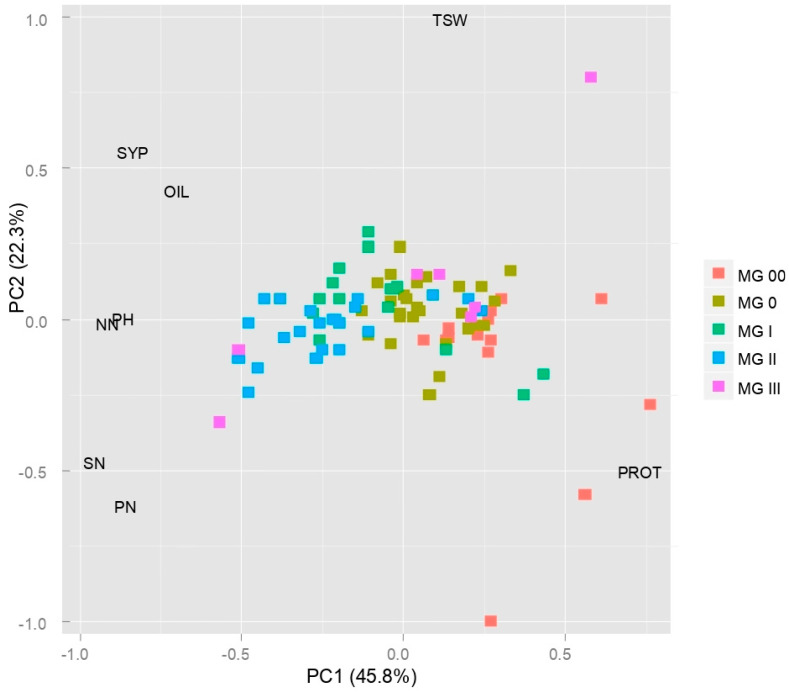
PCA biplot of 90 soybean genotypes based on 8 agronomic traits. Abbreviations: MG—maturity group; PH—plant height; NN—node number; PN—pod number; SN—seed number per plant; SYP—seed yield per plant; PROT—protein content; OIL—oil content.

PCA (Principal Component Analysis) of 90 soybean genotypes based on eight agronomic traits showed that the first two biplot axes accounted for 68.1% of the total phenotypic variability contained in the original data set (Figure 4). The majority of the genotypes assembled close to the origin, being intermediate for most of the traits observed. The grouping pattern displayed by the PCA biplot largely corresponded to the genotypes’ maturity classification. The following distribution of genotypes across the quadrants was observed: late-maturing (maturity group II) grouped in the lower left quadrant, mid-season (maturity group I) gathered in the upper right quadrant, very early (maturity group 00) predominantly allocated to the lower right quadrant, while early accessions (maturity group 0) mostly arranged around the biplot origin. Genotypes from maturity group III showed weak grouping consistency, intermingling with accessions from other maturity groups.

The biplot revealed that SYP—seed yield per plant, OIL—oil content, and the most important yield components (PH—plant height, NN—node number, PN—pod number, and SN—seed number per plant) discriminated genotypes along PC1, distinguishing those with high yield and high oil content on the left side of the biplot (MG I and MG II), and those with high protein content and low yield on the right (MG 00 and 0). The distribution of accessions by PROT—protein content was evident along PC1, with the highest number of positive extremes (four) observed in the group of very early genotypes (MG 00). The main contributor to the separation of genotypes along PC2 was TSW—1000-seed weight. A relative length of trait vectors suggested that TSW and SN were the major contributors to the total variation explained by the biplot (Figure 4).

#### 2.2.2. Cluster Analysis Based on Agronomic Traits

The phenotypic diversity of the 90 soybean genotypes based on eight agronomic traits was estimated by Euclidean distances (Table S3). The smallest Euclidean distance was determined for the pair Agassiz–Mini Soja (0.043) and the largest for the pair VNIMK 4895–PI 416 892 (1.200), while the average distance calculated was 0.244.

**Figure 5:**
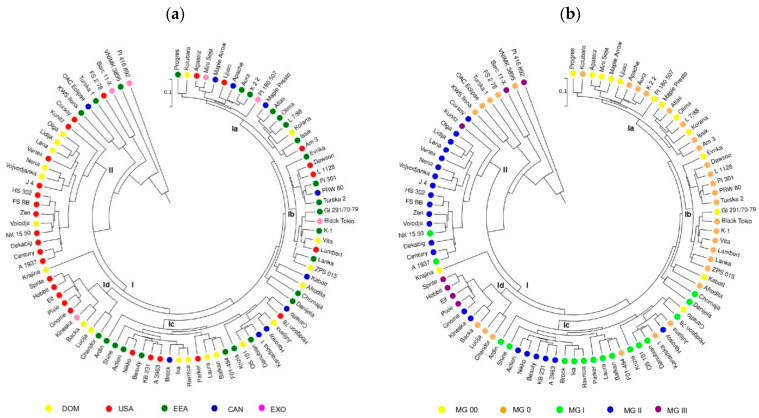
Dendrogram of UPGMA cluster analysis of 90 soybean genotypes based on 8 agronomic traits: (**a**) genotypes labelled by geographic origin: DOM—domestic; Serbia and Croatia; USA—United States of America; EEA—Europe and Euro-Asia; CAN—Canada; EXO—exotic; China and Japan; (**b**) genotypes labelled by maturity group (MG).

A UPGMA cluster analysis was performed to determine genetic relationships between the soybean genotypes by observing agronomic traits. There was no evident trend in the dendrogram’s structure indicating resemblance of genotypes from the same region (Figure 5a). The exceptions were noted for cluster II, where eleven American and six domestic genotypes clustered together, and cluster Id, which consisted predominantly of American accessions. On the other hand, the clustering reflected a clear maturity-related pattern of genotypes’ agglomeration (Figure 5b). The division of genotypes corresponded to the classification into early and medium–early (cluster I) and late varieties (cluster II). The dendrogram revealed four subclusters within cluster I: subcluster Ia (very early—MG 00 and early—MG 0 accessions), subcluster Ib (predominately MG 0 genotypes), subcluster Ic (mainly representatives of MG I), and subcluster Id (mainly late accessions). The genotypes of MG II were predominately allocated to cluster II, accounting for approximately 80% of this cluster.

### 2.3. Genetic Diversity as Indicated by SSR Markers

#### 2.3.1. SSR Polymorphism

All SSR primers generated clear DNA profiles of satisfactory band intensity. Among 21 primer pairs, 18 primers found to be polymorphic were subjected to further analysis. A total of 59 alleles were detected from these 18 primer pairs, of which 51 (86.44%) were polymorphic. The number of alleles generated from each primer pair ranged from two, obtained with Satt127, Satt406, Satt228, Satt308, Satt232, Satt172, Satt192, Satt225, Satt045, Satt191, Satt194, and Satt002, to six, generated with Satt114 (Figure S1). The average number of alleles per primer pair was 2.8.

#### 2.3.2. Cluster Analysis Based on SSR Markers

In order to assess genetic relationships among the 90 genotypes on the basis of SSR marker polymorphism, simple matching coefficients of similarity were calculated and transformed into genetic distances (Table S3). The lowest genetic distance was determined between the varieties Dawson and Harosoy (GD = 0.000), followed by the pair Turska 1 and Turska 2 (GD = 0.170); the largest genetic distance was observed for the Maple Arrow–Dekabig (GD = 0.627) pair, as well as for the PI 180 507–Am 3 and Kanadska 1–Gnome pairs (GD = 0.593). The average genetic distance among all genotype pairs was relatively low (GD = 0.317), reflecting the high genetic similarity in the experimental material.

**Figure 6:**
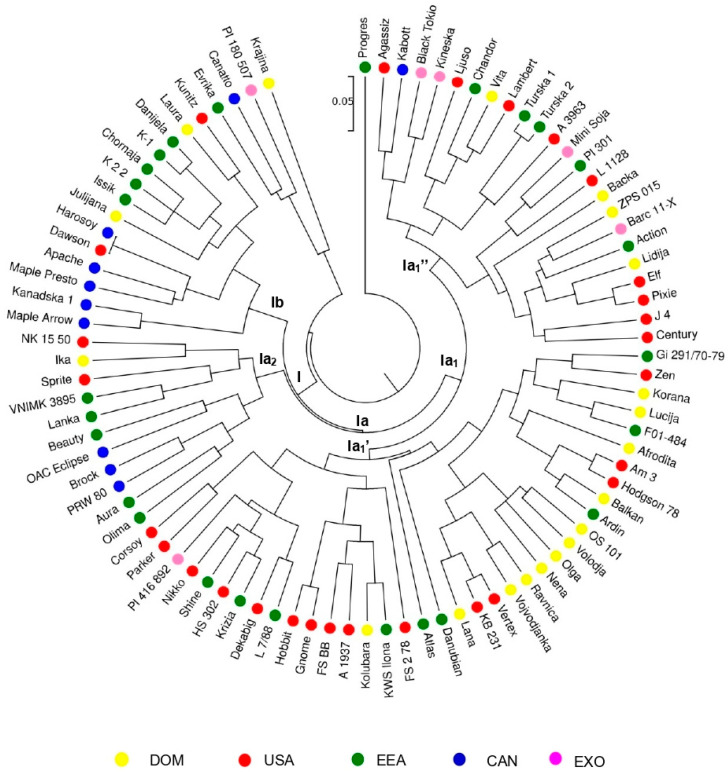
Dendrogram of UPGMA cluster analysis of 90 soybean genotypes based on SSR markers. Genotypes labelled by geographic origin: DOM– domestic; Serbia and Croatia; USA—United States of America; EEA—Europe and Euro-Asia; CAN—Canada; EXO—exotic; China and Japan.

A cluster analysis was performed to visualize genetic relationships among the genotypes according to the genotyping with 21 SSR primer pairs (Figure 6). The majority of genotypes were assigned to the main cluster (I), joined by a small group at a greater distance. The genotype Progres is considered separate from the main cluster. Cluster I is differentiated into two subclusters: Ia (encompassing Ia_1_ and Ia_2_) and Ib. Subcluster Ia_1_ is further divided into two groups—Ia_1′_ and Ia_1″_. Within the Ia_1′_ group, a smaller subgroup comprising ten American closely related cultivars could be observed. Among them, only two pairs of tightly linked pedigree-related varieties were noted: the cultivar Parker is related to the Corsoy variety through its descendants, and Hobbit and Gnome are the progeny from the same cross (Table S2). The larger subgroup of the Ia_1′_ group consisted of 21 genotypes, most of which (12) originated from Serbia and Croatia. This subgroup displayed a high consistency in genotype clustering with respect to the corresponding breeding program and partly agreed with the pedigree data: the cultivars Afrodita, Ravnica, Vojvođanka, F 01-484, and Hodgson 78 are descendants of the American variety Hodgson; the line OS 101 is the parental component of the cultivars Olga and Nena; and the varieties Lana and Vertex are indirect progeny of the American variety Williams. Pedigree data of the Romanian cultivars Danubian, Atlas, and F 014-484 revealed a common parent—the Tewels variety.

In group Ia_1″_, an intermingling of accessions from various geographic regions was observed, as well as the presence of a few pairs of genotypes with similar genetic backgrounds. The American cultivars Lambert and Agassiz, derived from the Evans variety, clustered together. The close grouping of four genotypes—Elf, Pixie, Lidija, and Barc 11-X—indicated their resemblance through the ancestral variety Williams. Two breeding lines (Turska 1 and Turska 2), which expressed considerable morphological similarity (Figure 1), appeared to be highly genetically related.

The division of 11 genotypes into small groups within subcluster Ia_2_ corresponded to the region of their origin. Three Canadian genotypes (OAC Eclipse, Brock, and PRW 80), two German genotypes (Aura and Olima), and two Russian genotypes (VNIMK 3895 and Lanka) were closely grouped. Pedigree data of the OAC Eclipse and Sprite varieties, which were positioned at a slightly greater distance but still in the same subcluster, confirmed their half-sib relation as direct descendants of the Williams variety.

Subcluster Ib included 14 genotypes, predominantly of Canadian (5) and Europe–Euro-Asian origin (5). Two Canadian cultivars—Apache and Maple Arrow—clustered near the Harosoy variety, which either directly or through its descendants contributed to their creation. The close grouping of the Maple Arrow and Maple Presto varieties was expected, considering that they represent half-sibs (common parent Swedish introduction Holmberg 840-7-3). The Bulgarian varieties Chornaja and Danijela fall into the same subgroup with genotypes from Kazakhstan (K-1, K 2 2, and Issik). The Kunitz variety, the parental component of the cultivar Laura, clustered in its close proximity. As disclosed by SSR analysis, the varieties Harosoy and Dawson appeared to be highly related (almost identical), which could be explained by the great contribution of Harosoy to the genetic background of Dawson.

#### 2.3.3. Principal Coordinate Analysis (PCoA)

**Figure 7:**
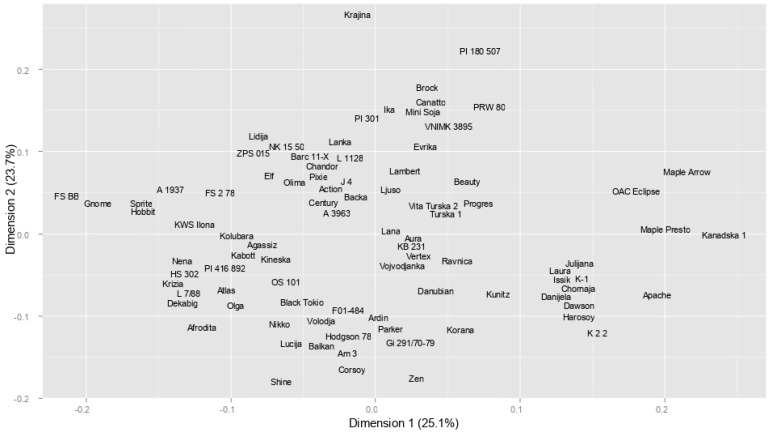
PCoA of the genetic structure of 90 soybean genotypes based on SSR markers.

The PCoA of the 90 soybean genotypes based on SSR markers showed that the first and second axes explained a total of 48.8% of the genetic variation contained in the original data set (Figure 7). The grouping pattern displayed by the PCoA plot highly corresponded to the grouping model derived by the cluster analysis. Groups composed of predominantly domestic, American, or Canadian genotypes that were identified in the cluster analysis were also noted on the PCoA graph. Pedigree-related genotypes that clustered closely showed a certain level of association on the PCoA plot as well. However, a few pairs of pedigree-related genotypes closely grouped in the cluster (Kunitz–Laura, or Afrodita–Vojvođanka and Afrodita–Ravnica) were separated on the PCoA graph, and vice versa—several pedigree-related genotypes separated in the cluster analysis were closely clustered on the diagram (Sprite–Hobbit and Sprite–Gnome).

#### 2.3.4. Analysis of Molecular Variance (AMOVA)

Analysis of molecular variance was applied to examine the distribution of genetic variation between and within five pre-defined geographic groups (Table 1 and Table S2). A statistically significant differentiation (*p* < 0.001) was observed between geographic groups; however, it accounted for just 6.1% of the total variation, while a considerably greater differentiation of 93.9% was attributed to within-group differences.

Pairwise comparisons of geographic groups were performed on the basis of the genetic differentiation index—Φ*_ST_*. A low pairwise differentiation between geographic groups (Φ*_ST_* ranged from 0.010 to 0.195) suggested a weak genetic differentiation and apparent gene flow. The lowest differentiation among all pairwise comparisons was observed between the DOM and USA groups (Φ*_ST_* = 0.01, *p* > 0.05), while the greatest differentiation was determined for Canadian vs. all other geographic groups (an average Φ*_ST_* = 0.148; *p* < 0.001). The EEA group did not significantly differentiate from all other groups except for CAN (Table 2).

### 2.4. Correlation Between Molecular and Phenotypic Variation

Mantel’s test was applied to assess the correlative relationships between dissimilarity matrices calculated from three data types. The correlation between SSR-generated and morphological descriptor-based distance matrices was positive and significant but still low (0.061, *p* < 0.05). A negative, weak, and non-significant association was determined between SSR-generated and agronomic trait-derived matrices (−0.027, *p* > 0.5), while the highest, but still low, degree of agreement was found between morphological descriptor-based and agronomic trait-derived matrices (0.179, *p* < 0.01). The observed correlations indicated that the variation in the phenotypic profiles of the examined genotypes was not associated with their genetic differentiation.

## 3. Discussion

### 3.1. Differentiation Pattern Revealed by Morphological Traits

The selected set of soybean genotypes displayed a relatively high morphological diversity, as indicated by the average genetic distance (GD = 0.517). The high morphological diversity of the examined set is shaped by the heterogeneous composition of the experimental material (released varieties, advanced breeding lines, and exotic germplasm), its wide geographic coverage, and the sufficient number of morphological descriptors used. However, a lower level of phenotypic variation estimated by DUS descriptors was reported in studies on registered varieties from commercial [32] and elite soybean pools [25].

In this study, HOMALS allowed for the identification of a minimum set of descriptors with the greatest discriminative power: hilum colour, seed coat colour, pubescence colour, and intensity of pod colour. The largest vectors observed for these traits indicated their great variability and, thus, their significant power to discriminate among genotypes. Descriptors of high discriminative power are important markers in germplasm evaluation and classification, diversity studies, variety identification, and the establishment of core collections [33]. Traits that efficiently discriminate between genotypes are also important in the system of variety registration and breeders’ rights protection, particularly in plant species with limited genetic variability, such as soybean [25]. The descriptor growth type expressed a moderate variation, given that the majority of the examined genotypes (73%) belong to an indeterminate class, which predominates in the growing regions of Europe, the USA, and Canada [20,34,35]. The rest of the evaluated descriptors exhibited a rather small variation, indicating their minor importance in distinguishing and identifying soybean genotypes. The flower colour and hypocotyl colour had identical coordinates in two-dimensional space, given that the inheritance of two traits is governed by a gene with a pleiotropic effect [36]. The grouping pattern displayed by clustering reflected the impact of different sets of descriptors (FC, PUBC, and GT) as compared to those identified by HOMALS. However, the genotypes’ allocation to the groups based on particular descriptors was less pronounced and more difficult to observe in the cluster analysis than in the HOMALS analysis.

Large soybean collections generally display clear geographic patterns of morphological differentiation [34,35], which was not the case in our research. Neither the cluster analysis nor the HOMALS analysis revealed consistency in the genotype’s grouping with respect to geographic origin. Similarly, Hegay et al. [37] discovered the absence of a geographic component of variation in a study of the morphological diversity of beans. The lack of association between descriptor-based grouping and the genotypes’ geographical origin might be primarily explained by the nature of the inheritance of the examined traits. Most of the observed descriptors are qualitative traits under monogenic or oligogenic control; the level of their expression is not influenced by environmental factors, and with the exception of HILC, SCC, PUBC, and FC, for which certain allelic interactions were determined [38], they mainly represent traits that are independently inherited and freely combined in various genetic backgrounds. Accordingly, the same structure of variation in qualitative morphological traits can be found in different genetic pools [39]. Furthermore, the descriptors approached in our study are not direct selection goals; they do not represent agronomically and technologically important traits and could be considered breeding-neutral characteristics that are randomly distributed among genotypes from different breeding programs and/or different geographical regions.

Generally, both applied multivariate methods displayed inconsistency in genotype grouping with respect to known pedigrees. Similar to Roldan-Ruiz et al. [24], the agreement between morphological similarity and pedigree relatedness referred only to individual pairs of genotypes but not to the groups as a whole. The absence of a consistent pedigree-related grouping determined in our study could be explained by the fact that the genotypes with a certain level of kinship were indirect descendants of parental genotypes included in our analysis, so their phenotypic profiles portray different combinations of morphological features of all progenitors that participated in their creation. While HOMALS accurately represented genetic relationships for only a few genotype pairs and one poorly consistent Williams-based group, the cluster analysis showed somewhat stronger consistency in the pedigree-related grouping, revealing a larger number of less consistent groups of related genotypes.

Although theory predicts complementarity between the two multivariate techniques [23], a low level of correspondence between two grouping patterns was evident and might be attributed to variation in the methods employed to analyse the same data set. HOMALS separated the genotypes into six clearly distinguished, moderately homogeneous groups, while the cluster analysis divided them into a greater number of less distant and more consistent groups. Given that successful classification implies high homogeneity, i.e., minimum variability within the same group, and high heterogeneity, i.e., maximum variability between different groups [40], HOMALS could be considered a more accurate and informative method for assessing diversity based on morphological descriptors.

### 3.2. Diversity Pattern Revealed by Agronomic Traits

The average Euclidean distance (0.244) indicated poor diversity of genotypes in terms of agronomic traits. Similar values of the average distances based on quantitative traits (0.417 and 0.321 for Chinese and North American accessions, respectively) were reported by Cui et al. [19], while Perić et al. [32] noted a somewhat higher diversity based on agronomic traits in a set of elite cultivars (0.481).

A moderate level of genotype dispersion on the PCA biplot confirmed rather low diversity considering eight quantitative traits. The first two biplot axes explained a relatively low portion of the total variation captured in the data set (68.1%), indicating complex relations among the traits observed [41]. It could be noted that PC1 clearly separated genotypes according to maturity group, despite the fact that the length of the vegetative period was not subjected to the analysis. Since Cui et al. [19] stated that the pleiotropic effect of maturity on yield-related traits introduces a small but significant bias effect in soybean cultivar comparisons, the variable “days to maturity” was excluded from our analysis.

When applied to soybean agronomic traits, multivariate methods mostly display the same grouping pattern—genotypes are assembled according to the maturity group [19,21,32,42,43]. The biplot revealed that the major drivers for the separation of genotypes along the PC1 axis were precisely the traits that are positively correlated with maturity (PH, NN, PN, SN, SYP, and OIL). Numerous authors confirmed the indirect impact of the photoperiod response on a wide range of soybean agronomic traits, such as plant height, node and pod number per plant, branching, protein and oil content, thousand seed weight, and maturity [44,45]. As maturity controls all stages of soybean plant development, differences in maturity between varieties are the best indicator of their phenotypic diversity [46]. Each developmental stage contributes to the formation of yield-related traits through various physiological mechanisms, determining the final yield [47]. The duration of the vegetative stage affects the production of total biomass [48], while the duration of the reproductive stage, consisting of flowering, pod formation, and seed filling, determines the formation of yield and its components [49], as well as the accumulation of seed protein and oil content [50]. Excluding the variable “days to maturity” from the analysis enabled an unbiased assessment of the grouping model and confirmed that the maturity-related differences generate all other variations in the agronomic performance of genotypes.

According to the UPGMA dendrogram, the grouping pattern based on agronomic traits was mainly inconsistent with data on geographic origin but exhibited considerable regularity regarding the maturity group. A similar trend was noted in studies employing multivariate methodology to examine the diversity of soybean agronomic traits [51,52,53], where the geographical components of phenotypic diversity could not be observed despite the theoretical expectation that the agronomic profiles of varieties reflect their geographic origin. Adaptation to the environmental conditions in a certain geographical area, primarily the photoperiod length and the temperature sum [19,54], as well as region-specific breeding goals [55], significantly contribute to the phenotypic differentiation of genotypes from various regions. Despite the inconsistency in grouping with respect to geographic regions, the clustering displayed a clear phenotypic distinction of genotypes from different maturity groups. The absence of a geographical pattern of variation, on the one hand, and a clear maturity-related grouping, on the other, could be explained by the criteria for experimental set selection. Despite the fact that the evaluated genotypes were primarily chosen to represent diverse geographical regions, they had to meet the criterion of adaptation to local agro-ecological conditions, which narrowed the choice to genotypes covering only five MGs (00-III). Although geographically distinct, the observed regions do not, at least not in terms of photoperiod length, represent ecologically contrasting environments. Had we employed late-maturing varieties from South America, the results may have been different. Additionally, regional differences were probably not discernible because the representatives in each geographical group varied greatly in maturity, with the exception of the Canadian group, which spanned only two maturity groups (MG 00 and 0).

### 3.3. Genetic Diversity as Indicated by SSR Markers

High values of SSR marker polymorphism (over 70%) are reported by numerous studies [56,57,58], confirming that SSRs are informative and an effective tool for soybean diversity assessment. Similar to our results, a low average number of alleles per primer (less than five) was reported in studies of SSR polymorphism in soybean local collections [29,56,58,59], while a slightly greater average number of alleles per primer (5–6) was found in assessments of commercial and/or elite material [30,60,61]. Asian soybean collections reflected the highest diversity, with an average number of alleles per primer of 11–14 [20,62,63].

A low level of genetic diversity indicated by genetic distances (GDs < 0.3) was confirmed in numerous studies of soybean SSR polymorphism in small regional collections [58,59] or sets of local commercial varieties [64,65,66]. The highest average distances (GD > 0.6) were reported in analyses of diverse material from large Asian germplasm collections [62,67] and exclusively in commercial material from Argentina [25]. As a general trend, soybean populations derived from biparental crosses of genetically distant parents are more likely to exhibit a higher genetic variance for important breeding-related traits than the populations derived from crosses of genetically related ones. Genetically distant pairs of accessions (i.e., Maple Arrow–Dekabig, PI 180 507–Am 3, and Kanadska 1–Gnome) that were identified within the experimental set could serve as potential parental components in future breeding programs.

Although numerous studies reported the high-resolution power of SSR markers [68], the present study yielded somewhat different results. A notable example is the Harosoy–Dawson genotype pair, where SSR markers failed to detect differences at the genome level despite significant phenotypic distinctness, as indicated by 15 morphological (GD = 0.313) and eight agronomic (GD = 0.178) traits. However, several genotype pairs (Apache–Harosoy, Bačka–Kolubara, Harosoy–KB 231) with almost identical morphological profiles (GD = 0.063) expressed considerable diversity at the molecular level.

Similar to previous assessments of soybean SSR diversity [59,66,69], the grouping pattern revealed by the cluster analysis reflected a moderate consistency in the clustering with respect to pedigree, which mainly referred to individual pairs or smaller groups of genotypes within the subclusters but not to the groups as a whole. A weakly pronounced pedigree-based grouping was unsurprising due to the limited coverage of the soybean genome assessed with a relatively small number of SSRs (21) and given that certain accessions in the experimental set had incomplete pedigree data. Priolli et al. [70] found a low but significant correlation between SSR-derived similarity indices and kinship coefficients, concluding that the mentioned parameters could be relevant indicators of genetic diversity only in the case of complete pedigree data and a sufficient number of markers. Furthermore, a similar parental combination does not necessarily imply similarity in the progeny since the continuous process of inbreeding, combined with phenotypic selection, can result in a different proportion of the parental genome in the genetic background of offspring [25].

The literature findings regarding the molecular diversity of soybean germplasm indicated that the grouping pattern is dominated by the geographical component of molecular diversity, i.e., the tendency of genotypes from the same geographical region to cluster into more or less homogeneous groups [30,59,61,71]. In the present study, although no clear regional differentiation between major cluster groups was detected, several minor groups had a greater proportion of genotypes from the same geographical region and/or particular breeding program. Canadian varieties tended to cluster together (clusters Ia_2_ and Ib) and were mostly surrounded by European–Euro-Asian genotypes. This clustering model partially agrees with the results of Fu et al. [66], who determined, by SSR analysis, that Canadian varieties were more related to germplasm from North–Eastern Europe (Russia, Sweden, and Ukraine) than to Asian introductions. Additionally, the clustering of German cultivars with Canadian ones is in accordance with the findings of Saleem et al. [10], who revealed a considerable relatedness between Western European and Canadian accessions. The most prominent regional separation was observed for two subgroups within cluster Ia_1′_, which clearly distinguished American genotypes from Serbian and Croatian varieties. The association of American genotypes in the same subcluster with Serbian was somewhat expected, given that the majority of Serbian elite varieties traced back to American introductions [29]. In parallel, the high genetic similarity in elite varieties developed in Western Balkans breeding programs (Serbia and Croatia) was already reported by Perić et al. [32]. This grouping trend is the result of the limited exploitation of the small proportion of genetic resources available in local collections, as well as the excessive use of crosses between elite lines in local breeding programs, which were created through a long process of phenotypic selection, adapted to the specific regional conditions, and thus fairly genetically uniform [11]. This confirms the conclusion of Qiu et al. [54] that the greatest selection influence in the natural and artificial selection of soybean was attributed to the photoperiod length and the temperature sum, which greatly vary in different areas of soybean cultivation and are a key factor in the adaptation of genotypes to specific regional conditions, the creation of their genetic and phenotypic structure, and the differentiation of genotypes in relation to geographic origin [72].

In line with previously published findings [37,73], the PCoA grouping pattern showed a high degree of agreement with the cluster-based grouping, both reflecting the geographical distribution of molecular diversity previously determined [66,67,74]. Pedigree-related genotypes that closely clustered on the dendrogram exhibited a certain level of association in the PCoA as well, with some minor inconsistencies observed. Messmer et al. [75] stated that cluster analysis was more sensitive and thus more reliable than PCoA for discovering pedigree relations among genotypes when the first two axes explain less than 25% of the total variation. Our results might corroborate the latter findings, given that the first two axes of the PCoA captured a relatively low portion of the total variation (<50%).

Identifying the model of the ecogeographical distribution of genetic variation in a given collection allows for the geographic region to be used as a criterion for the core set’s creation, resulting in improved resource management and usage [76]. The AMOVA revealed that just a small portion of the total variation was attributed to between-group variation (6.1%), indicating a weak regional differentiation of the examined set. Several studies revealed that variation between geographic groups accounted for less than 10% of the total variance [29,66,71,77]. Tavaud-Pirra et al. [52] examined five geographic groups within the INRA soybean collection using SSR markers and determined that the genetic structure of the collection did not correlate with geographic origin (Φ*_ST_* values ranged from 0.002 to 0.04). A low level of differentiation of our experimental set might be due to the fact that we assessed a relatively small number of highly pedigree-related genotypes with narrow regional adaptation. Although some clusters shared common parents, the main groups could not be clearly traced back to origin-related geographic regions.

The highest level of differentiation in relation to all the examined groups was observed for Canadian genotypes. These cultivars are unique in their geographic and climatic adaptation (higher latitudes and cooler climates), covering a narrow range of maturity groups [65,66]; thus, they might have had quite different genetic structures compared to representatives of other geographical groups, which are mainly characterized by later maturity. As indicated by Φ*_ST_*, Canadian genotypes were more closely related to the EEA group than to EXO [66,78] or to USA accessions [78].

The domestic varieties showed the lowest degree of differentiation compared to the USA group, confirming the predominant genetic contribution of the American-introduced lines from the Northern collection of soybean germplasm in the genome of domestic varieties [29,79]. Our findings are consistent with Andrijanić et al. [71], who reported a rather low differentiation between USA and Serbian (Φ*_ST_* = 0.06) and USA and Croatian genotypes (Φ*_ST_* = 0.11). Additionally, the DOM group did not differentiate significantly from the EEA group, which indicates the existence of an exchange of genetic materials within the broad region of Europe.

A low level of diversity between EEA and USA genotypes was expected given that the EEA group is large (26 accessions), geographically and genetically heterogeneous, and presumably captures significant within-group variation. Furthermore, the notably low mean differentiation between American and European genotypes reported by Andrijanić et al. [71] supported the findings about the frequent use of American material in European soybean breeding [30,32,52,59]. The EEA group was also weakly differentiated from the small group of Asian accessions. Since the EEA group’s composition revealed that half of the accessions came from Eastern Europe (Poland, Bulgaria, Romania, Russia, and Kazakhstan), our results are consistent with those of Saleem et al. [10], who reported that Chinese diversity was significantly incorporated into the genetic background of Eastern European genotypes. An additional contribution to the absence of differentiation between the EXO and EEA groups can be attributed to Russian and Kazakh varieties, for which a strong genetic relationship with the North Chinese genetic pool has been reported [80].

### 3.4. Comparision of Genetic Distances Generated from Three Data Types

The average genetic distances based on three marker types indicated that the examined genotypes expressed a low variation in agronomic traits (0.244), slightly higher molecular diversity (0.317), and considerable variation in morphological profiles (0.517). The lowest diversity of soybean genotypes with regard to agronomic traits was somewhat expected considering the relatively small number of highly correlated traits analysed, as well as the narrow range of maturity groups covered by the experimental set. Surprisingly, the diversity estimated by morphological descriptors was greater than the diversity at the molecular level. The descriptors employed in this study are part of the DUS protocol for the identification and differentiation of soybean cultivars and are assumed to exhibit high polymorphism. Furthermore, besides the varieties and breeding lines, the experimental set included germplasm with specific morphology, which likely contributed to the high diversity.

The correlative relationships between genetic and phenotypic similarity indices were assessed by the application of Mantel’s test [81]. Determining the positive correlation of two distance matrices enables the efficient use of morphological traits in the routine evaluation of breeding material without the necessary use of laboratory methods [82].

A positive and statistically significant (*p* < 0.05) correlation between morphological descriptor-based and SSR-generated distance matrices could have led to the conclusion that genotypes with similar SSR profiles expressed a certain morphological similarity. However, the correlation coefficient was apparently very low (r = 0.061), matching with the results reported in studies of soybean [25] and other self-pollinated legumes [26,37,83]. Our results implied that estimates based on morphological markers can be considered, at best, indirect measures of genetic diversity [25]. A previous author emphasized that merging quantitative and qualitative data into the same set for multivariate analysis, as a frequently encountered approach in the studies of phenotypic diversity [28,84], may yield significant bias in GD estimates and reduce the reliability of the derived correlation coefficients. Consequently, the correlation found in the present study was considered a fairly reliable indicator of the molecular and morphological association since we performed a separate analysis for two data sets.

Mantel’s test determined a negative, low, and non-significant (*p* < 0.05) correlation between SSR-generated and agronomic trait-derived distance matrices, suggesting that agronomic traits cannot be considered reliable indicators of genetic diversity. Microsatellites reflect genetic variation in coding and non-coding regions of DNA and are not always linked to genes governing quantitative traits [37]. A strong association between the two matrix types can be expected in the case of the presence of a link between the SSR marker and the quantitative traits loci (linkage disequilibrium), especially if the two genotypes are similar in pedigree [85]. Furthermore, the lack of association between two matrix types can be attributed to the complex inheritance of quantitative traits, whose expression depends on a large number of genes and their interactions and is strongly influenced by environmental factors [86]. The high correlation between the distances estimated by the mentioned marker types might be a signal of a limited genetic pool because such genotypes likely share the same genetic background and/or originate from programs with identical breeding goals [24].

A highly significant (*p* < 0.01), low, and positive association between the distance matrices derived from morphological descriptors and agronomic traits suggested that genotypes with similar combinations of morphological descriptors exhibited a similar agronomic performance as well. Such results were not expected, considering the assumed independence between morphological descriptors and agronomic traits. The only exception might be attributed to the descriptor growth type, which possibly had an indirect effect on yield components [87,88]; nevertheless, the correlation was rather weak, indicating that the influence of the 15 other descriptors outweighed the effects of the mentioned one.

## 4. Materials and Methods

### 4.1. Plant Material

A set comprising 90 accessions was selected from the MRIZP soybean collection, considering the criteria of good adaptive ability to local agro-ecological conditions and potential relevance as a breeding source. The representativeness of the experimental set, i.e., reliable representation of the geographical diversity of the collection, was achieved by selecting genotypes from different geographical regions in proportion to their relative contribution to the collection. Genotypes were classified into 5 geographic groups, which corresponded to the region of their origin: DOM—domestic; Serbia and Croatia (20), USA—United States of America (28), EEA—Europe and Euro-Asia (26), CAN—Canada (10), EXO—exotic; China and Japan (6). The examined genotypes belong to various maturity groups (MG 00—III) and are designated by different collection statuses (released varieties, advanced breeding lines, germplasm with specific morphology). Detailed information on plant material, including MG, origin, known pedigree, and collection status, is summarized in Table S2.

### 4.2. Field Trial and Phenotyping

The experimental material was classified according to data on maturity recorded in Serbian agro-climatic conditions, which closely matched the information on maturity from the region of origin. Five field experimental sets were established, with varying numbers of representatives from each maturity group: MG 0 (very early)—14; MG 00 (early)—31; MG I (mid-early)—16; MG II (late)—22; and MG III (very late)—7. A two-year field experiment (2021 and 2022) was conducted at two locations in Serbia (Zemun Polje and Pančevo), designed according to a randomized complete block design with 3 replications. Each genotype was sown in two 5 m rows with an inter-row distance of 0.5 m, creating an experimental plot of 5 m^2^. Two rows of isolation were sown between the plots to eliminate the competition effect. Standard agricultural practice for soybean cultivation was applied. The soil type was non-carbonate chernozem at Zemun Polje, i.e., carbonate chernozem on the loess terrace at Pančevo.

The morphological description was carried out by observing fifteen selected morphological traits (descriptors), following the UPOV technical guidelines for the conduct of tests for distinctness, uniformity, and stability (DUS) for soybeans [89]. The observed descriptors, level of phenotypic expression (category), level scores, and frequency distribution across genotypes are presented in Table S4. Plant morphological traits for each genotype were assessed visually by observing a group of plants in the field, while the seed traits were evaluated in the laboratory after harvesting and manual threshing of the samples. Descriptor scores were moderately modified with respect to technical guidelines: for binomial features, assigned values were 1 and 2, while multinomial data were scored with odd numbers from 1 to n. An additional modification was applied to the descriptor “growth type” by establishing the category “fasciata”, which does not appear in the UPOV standard due to the absence of the aforementioned growth type in the commercial material.

The field experimental sets were evaluated for eight quantitative variables, encompassing both yield-related and quality-related traits, which we classified as agronomic for the clarity of interpretation. At the R8 growth stage [90], 25 plants of each genotype per replication were randomly selected and scored for plant height—PH (cm), node number—NN, and pod number—PN. Individual plants were threshed manually before recording seed yield per plant—SYP (g), seed number per plant—SN, and 1000-seed weight—TSW (g). After completing the measurements, the seeds of individual plants of each genotype per replication were mixed in samples (200 g) for analysis of seed protein—PROT (%) and oil—OIL (%) content (expressed on a dry matter basis) using a near-infrared reflectance-based grain analyser from Infraneo Chopin Technologies^®^, (Chopin Technologies, Villeneuve La Garenne, France).

### 4.3. DNA Extraction and Genotyping

The genomic DNA was isolated from the seeds of 1 plant per genotype, according to the method of [91]. A set of 21 commercial microsatellite markers (SSR) produced by Metabion Int. (Table S5) was selected to cover each linkage group based on the genetic map by Song et al. [92]. The reaction mixture for DNA amplification consisted of 1 unit of Taq polymerase, 1× PCR buffer (DreamTaq™ Green Buffer, Thermo Fisher Scientific Inc., Waltham, MA, USA), 2.4 mM MgCl2, 0.8 mMdNTP, 0.5 µM primers (forward and reverse), 50 ng/µL genomic DNA, and nuclease-free H_2_O made up to a volume of 25 µL. Polymerase Chain Reaction (PCR) was carried out in a Professional Standard Thermocycler (Biometra) device, applying the Touchdown program. Initial denaturation was performed at 95 °C for 5 min, followed by 15 cycles of denaturation at 95 °C for 30 s, annealing at temperatures in a range from 56 °C to 63.5 °C (depending on the primer pair used) for one minute (with a decrease of 0.5 °C per cycle), and extension at 72 °C. The reaction was completed by 25 cycles of denaturation at 95 °C for 30 s, annealing at 56 °C for 1 min, and elongation at 72 °C for 1 min. Amplification products were separated by electrophoresis on an 8% polyacrylamide gel in a four-chamber system Mini Protean Tetra-Cell (BioRad, Bio-Rad Laboratories, Inc., Berkeley, CA, USA). A 100 bp DNA ladder (Thermo Fisher Scientific Inc., Waltham, MA, USA) was used as a molecular weight marker. Post-separation, the gels were stained with ethidium bromide for 30 min and then imaged under UV light using the Biometra BioDocAnalyze Live gel documentation system. The presence or absence of DNA bands was transformed into binary data (1 and 0, respectively).

### 4.4. Statistical Analysis

The morphological diversity of soybean genotypes was assessed by application of HOMALS—Homogeneity Analysis by Means of Alternating Least Squares, otherwise known as factorial analysis for categorical data [93]. Categories arrange objects into homogeneous groups, ensuring that each object is close to the point representing a specific object’s category. The discriminative power of a descriptor represents the variance in a quantified descriptor along a given dimension [94]. A strong discriminative power corresponds to a large dispersion of categories and vectors. HOMALS analysis was performed using the R statistical package [95]. A morphological distance matrix between pairs of genotypes was generated by calculating the proportion of common nominal categories of descriptors and further employed for UPGMA (unweighted pair group method with arithmetic mean) clustering using NTSYS-pc [96]. Given that each genotype has a single nominal category for a certain descriptor, the distance calculated using the proportions of shared categories is identical to the distance calculated by the simple matching coefficient [97].

The pattern of genotype grouping according to the similarity in the agronomic traits was analysed by two multivariate methods—PCA (Principal Component Analysis), using the R package [95], and UPGMA non-hierarchical cluster analysis, performed in NTSYS-pc [96]. The relatedness of genotypes based on eight agronomic traits was visualized by a dendrogram constructed on the basis of the Euclidean geometric distances matrix [98]. Prior to this, each trait was normalized in relation to the genotype with the maximum score, ensuring the values of the agronomic traits in the data matrix ranged in the interval 0–1.

Data generated by SSR analysis were used to calculate simple matching coefficients of similarity [97], which were further converted to genetic distances (GDs) as described by Reif et al. [99]. The GD matrix was subjected to cluster analysis using the UPGMA algorithm for constructing genotype groups in NTSYS-pc [96], and principal coordinate analysis (PCoA) was used to reduce the matrix dimensions into several interpretive principal coordinates, computed in R software [95]. Arlequin 2.0 software [100] was used for the analysis of molecular variance (AMOVA) [101] in order to estimate the magnitude of within- and among-group differentiation following the previously defined geographical groups. Pairwise comparisons were performed on the basis of Φ*_ST_*, which represents the standardized inter-population distance between two geographic groups, as measured by the correlation between genes of different individuals in one population [76]. According to Hartl and Clark [102], differentiation was considered weak (Φ*_ST_* < 0.05), moderate (0.05 < Φ*_ST_* < 0.15), large (0.15 < Φ*_ST_* < 0.25), and very large (Φ*_ST_* > 0.25). The agreement between distance matrices generated from three data types was estimated by Mantel’s test [81]. The trees were constructed and visualized using MEGA software [103].

## 5. Conclusions

A comprehensive study of the MRIZP soybean working collection indicated considerable diversity on an agro-morphological level, reflected through the variety of phenotypic profiles of the studied accessions. From the breeder’s viewpoint, information generated from phenotypic data remains the most relevant guideline for successful germplasm management and different breeding objective achievement. However, the lower level of diversity estimated by SSRs should be viewed as conditional due to the relatively small number of markers employed in the analysis. The latter does not diminish the scientific and practical significance of our findings, as the complementary method (i.e., simultaneous evaluation of morphological and agronomic traits) buffers the possible bias associated with relying on a limited number of SSR markers. All of these findings contributed to a better understanding of the significance of local small working collections, which encompass adapted germplasm created by pre-breeding activities and emphasized their valuable applicability as excellent sources for achieving various breeding goals. As a long-term consideration for continuous progress in soybean breeding, actively enlarging working collections and maintaining genetic diversity enables the development of more sophisticated breeding strategies, including marker-assisted selection and genomic selection, to speed up the breeding process and improve the efficiency of developing new soybean varieties.

## Figures and Tables

**Table 1:** Analysis of Molecular Variance (AMOVA) for 90 soybean genotypes among 5 geographic groups based on SSR polymorphism.

Source of Variation	d.f.	Variance Component	%	Φ*_ST_* ^#^	*p* ^#^
Between Groups	4	0.008	6.1	0.061	0.000
Within Groups	85	0.125	93.9		

# calculated based on 1000 permutations.

**Table 2:** Pairwise comparisons of geographic groups based on Φ*_ST_* values from AMOVA.

Group Pairs	Source of Variation	d.f.	Variance Component	%	Φ*_ST_*	*p* ^#^
CAN vs. DOM	Between groups	1	0.028	19.2	0.192	0.000
	Within groups	28	0.119	80.8		
CAN vs. EEA	Between groups	1	0.013	9.2	0.091	0.002
	Within groups	34	0.133	90.8		
CAN vs. EXO	Between groups	1	0.016	11.5	0.115	0.046
	Within groups	14	0.129	88.5		
CAN vs. USA	Between groups	1	0.028	19.5	0.195	0.000
	Within groups	36	0.119	80.5		
DOM vs. EEA	Between groups	1	0.003	2.7	0.027	0.053
	Within groups	44	0.129	97.3		
DOM vs. EGZ	Between groups	1	0.013	9.5	0.094	0.018
	Within groups	24	0.123	90.5		
DOM vs. USA	Between groups	1	0.001	1.0	0.010	0.236
	Within groups	46	0.118	99.0		
EEA vs. EXO	Between groups	1	0.002	1.7	0.016	0.262
	Within groups	30	0.137	98.3		
EEA vs. USA	Between groups	1	0.002	1.8	0.017	0.103
	Within groups	52	0.127	98.2		
EXO vs. USA	Between groups	1	0.002	2.1	0.021	0.226
	Within groups	32	0.122	97.9		

# calculated based on 1000 permutations; *p* < 0.001—highly statistically significant; *p* < 0.05—statistically significant.

## Data Availability

The data are contained within this article or Supplementary Materials.

## Supplementary Materials

The following supporting information can be downloaded at https://www.mdpi.com/article/10.3390/plants14020201/s1. Figure S1: DNA profile obtained with Satt114 marker; Table S1: Visual scores for 15 morphological descriptors in 90 soybean genotypes, Table S2: List of soybean genotypes, collection status, maturity group, breeding institution, country of origin, geographic region and pedigree; Table S3: Genetic distances generated from three marker types; Table S4: List of descriptors, categories, category scores, and distribution of genotypes across descriptor categories; Table S5: Sequences of 21 SSR primers used in the analysis.
