# Supplementary material for: Depicting Soybean Diversity via Complementary Application of Three Marker Types
Source: Plants (Basel). 2025 Jan 12;14(2):201. doi: 10.3390/plants14020201 (PMC11768110; doi:10.3390/plants14020201)
Supplement: Supplementary file 1 [file plants-14-00201-s001.zip › Table S1.pdf]

Table S1. Visual scores for 15 morphological descriptors in 90 soybean genotypes

**No.**	**Genotype**	**HIPC**	**H**	**GT**	**PUBC**	**LB**	**LLSh**	**LLS**	**ILC**	**FC**	**IPC**	**SSh**	**SCC**	**SL**	**HILC**	**CHF**
1	Progres	1	1	7	1	5	5	1	3	1	1	5	1	1	7	2
2	Agassiz	1	1	7	2	5	5	1	1	1	3	1	1	2	3	1
3	Evrika	2	3	7	2	5	7	1	3	2	1	1	1	2	1	1
4	Gi 291/70-79	2	3	7	1	5	5	3	1	2	3	1	3	1	7	2
5	Kabott	1	3	7	2	7	7	3	3	1	1	1	1	1	1	1
6	Ljuso	1	3	5	1	7	7	3	1	1	3	3	1	1	5	1
7	Maple Arrow	1	1	7	1	5	5	1	3	1	5	3	1	2	7	1
8	Maple Presto	1	1	7	1	5	7	1	3	1	5	3	3	2	7	1
9	Mini Soja	2	1	7	2	5	5	1	5	2	1	3	1	1	1	1
10	Canatto	2	1	7	2	5	7	1	1	2	3	3	1	1	1	1
11	PI 180 507	2	3	7	1	7	5	3	3	2	3	7	1	1	5	1
12	Korana	1	3	7	1	7	7	3	3	1	5	7	1	2	7	2
13	Olima	2	3	5	1	7	7	1	3	2	3	1	1	1	7	2
14	Krajina	1	3	7	2	5	7	3	3	1	1	3	1	2	1	1
15	FS 2 78	1	3	5	1	5	5	1	3	1	3	3	3	2	7	2
16	Afrodita	1	3	7	2	3	5	3	3	1	1	3	1	2	1	1
17	Apache	1	3	7	2	7	5	3	1	1	3	3	1	2	1	1
18	Atlas	2	3	7	2	5	5	3	5	2	3	1	1	1	1	1
19	Aura	2	3	7	2	5	5	1	5	2	1	1	1	1	3	2
20	Black Tokio	2	1	7	1	7	7	5	3	2	5	3	7	1	15	1
21	Chandor	2	3	5	1	3	7	3	3	2	1	5	5	2	13	1
22	Dawson	1	3	7	2	5	5	1	3	1	3	5	3	2	1	1
23	Lucija	1	1	3	1	5	7	3	3	1	5	3	1	1	11	1
24	F01-484	1	5	7	2	5	5	5	3	1	5	3	1	2	3	1
25	Julijana	1	1	7	2	5	7	5	3	1	1	3	1	1	13	1
26	Backa	1	1	7	2	5	7	3	3	1	3	3	1	2	1	1
27	Issik	2	3	5	2	5	7	3	3	2	3	1	3	2	17	1
28	K-1	1	1	7	1	3	5	1	1	1	3	5	1	2	7	1
29	K 2 2	2	1	1	2	5	7	5	3	2	3	3	1	1	1	1
30	Kanadska 1	2	3	7	1	5	7	1	1	2	3	1	5	1	5	1
31	L 1128	2	3	5	1	5	5	1	3	2	3	1	1	2	7	1
32	KWS Ilona	1	1	7	2	3	7	1	3	1	1	3	1	2	13	1
33	Vita	1	5	7	2	5	5	3	3	1	5	3	1	1	9	1
34	Lambert	1	5	7	2	3	5	1	3	1	3	5	1	1	3	1
35	Lanka	1	3	3	2	7	7	5	3	1	1	5	1	1	11	1
36	OAC Eclipse	1	5	5	1	5	7	3	3	1	5	5	1	1	5	1
37	L 7/88	1	3	3	1	7	5	3	5	1	5	5	3	2	5	2
38	PI 301	1	1	7	2	5	5	3	5	1	1	3	1	2	1	1
39	PRW 80	1	3	7	1	5	7	3	3	1	5	7	1	1	5	1
40	VNIMK 3895	2	1	1	1	5	5	5	3	2	5	7	1	1	5	2
41	Turska 1	2	5	7	2	3	7	1	3	2	1	1	1	2	1	1
42	Turska 2	2	5	7	2	3	7	1	3	2	1	1	1	2	5	2
43	Am 3	1	1	7	2	3	5	1	5	1	1	1	1	2	1	1
44	ZPS 015	1	1	7	1	3	5	5	3	1	3	3	5	2	11	1
45	Kolubara	1	1	7	2	5	7	3	3	1	3	3	1	2	5	1
46	Balkan	2	1	7	2	5	5	5	5	2	1	3	1	2	3	1
47	Chornaja	2	5	7	1	5	7	5	1	2	5	3	7	2	15	1
48	Danijela	1	5	1	1	7	7	5	5	1	5	7	9	1	7	1
49	Danubian	2	3	5	1	9	7	5	3	2	5	5	1	1	15	1
50	Hodgson 78	1	3	7	2	3	5	3	5	1	5	5	1	2	5	1
51	Ardin	1	7	7	2	7	5	3	5	1	1	1	1	1	1	1
52	Krizia	1	3	5	1	3	5	3	1	1	5	3	1	2	7	2
53	OS 101	1	3	7	2	5	7	5	3	1	1	7	1	1	3	1
54	Ravnica	1	5	7	2	3	5	5	5	1	5	1	1	2	1	1
55	Ika	1	3	7	2	3	7	3	3	1	5	3	3	2	1	1
56	Shine	2	3	7	2	5	1	1	5	2	1	5	1	2	3	1
57	A 1937	1	3	7	1	5	7	3	5	1	5	5	1	2	3	1
58	Laura	2	3	7	1	5	5	5	3	2	1	7	1	1	15	1

Table S1. (continued)

No.	Genotype	HIPC	H	GT	PUBC	LB	LLSh	LLS	ILC	FC	IPC	SSh	SCC	SL	HILC	CHF
59	NK 15 50	1	3	7	2	5	5	5	5	1	3	5	1	2	11	1
60	Parker	2	3	3	2	5	5	3	5	2	3	5	1	2	3	1
61	Brock	1	3	7	1	5	5	5	1	1	3	5	3	1	5	1
62	A 3963	1	1	7	1	5	7	3	3	1	5	3	1	2	3	2
63	Dekabig	1	3	7	1	7	7	3	3	1	5	3	1	2	5	1
64	FS BB	1	3	7	1	5	5	5	5	1	1	3	1	2	15	1
65	Gnome	1	3	1	1	9	7	5	5	1	3	5	1	1	15	2
66	Harosoy	1	3	7	2	7	5	3	5	1	3	3	1	2	1	1
67	HS 302	1	1	7	1	7	7	5	5	1	5	3	1	2	1	1
68	Kineska	2	1	1	2	7	7	5	3	2	1	1	1	2	9	1
69	Nikko	1	5	7	1	7	7	5	5	1	5	3	1	2	5	1
70	Action	2	3	7	2	5	7	1	5	2	1	3	1	1	9	1
71	Beauty	1	3	7	2	5	5	3	3	1	1	7	1	2	1	1
72	J 4	1	5	7	1	5	5	5	3	1	3	3	1	2	15	1
73	Vertex	1	1	7	2	5	5	5	5	1	5	5	1	2	5	1
74	Volođa	1	3	7	2	5	7	3	3	1	1	5	1	1	3	1
75	Vojvođanka	1	3	7	2	5	7	5	5	1	5	3	1	2	1	1
76	Lana	2	1	7	2	5	5	5	3	2	5	7	1	1	3	1
77	Olga	1	3	7	1	5	5	3	3	1	3	3	1	2	7	1
78	Lidija	2	1	7	2	5	5	5	3	2	5	3	1	1	1	1
79	Nena	1	3	7	1	5	5	5	3	1	5	3	1	1	5	1
80	Zen	1	3	7	1	5	5	3	5	1	5	7	1	2	15	1
81	Century	1	3	7	1	5	5	3	3	1	1	3	3	2	15	2
82	Corsoy	1	1	7	2	5	5	5	1	1	3	7	1	1	1	1
83	KB 231	1	1	7	2	7	5	3	5	1	3	3	1	2	1	1
84	Elf	1	3	1	1	7	5	5	5	1	3	7	1	1	15	1
85	Hobbit	2	3	1	1	5	5	5	5	2	1	5	1	1	15	1
86	Kunitz	2	5	7	1	5	7	5	1	2	3	7	1	1	15	1
87	Sprite	2	3	1	1	3	5	5	5	2	3	3	1	1	15	2
88	PI 416 892	1	7	3	1	9	7	5	1	1	5	7	7	1	15	2
89	Barc 11-X	2	3	9	1	7	7	5	5	2	3	3	5	1	15	1
90	Pixie	1	3	1	1	5	7	5	5	1	3	3	3	1	15	1

HIPC – hypocotil color; **H –** habitus; **GT –** growth type; **PUBC** – pubescence color; **LB** – leaf blistering; **LLSh –** shape of lateral leaflet; **LLS –** size of lateral leaflet; **ILC –** intensity of the green color of the leaf; **FC –** flower color; **IPC –** intensity of the brown color of the pod; **SSh –** seed shape; **SCC** – seed coat color; **SL** – seed coat luster; **HILC –** hilum color; **CHF** – color of hilum funicle.
