# Supplementary material for: Depicting Soybean Diversity via Complementary Application of Three Marker Types
Source: Plants (Basel). 2025 Jan 12;14(2):201. doi: 10.3390/plants14020201 (PMC11768110; doi:10.3390/plants14020201)
Supplement: Supplementary file 1 [file plants-14-00201-s001.zip › Table S2.pdf]

Table S2. List of soybean genotypes, collection status, maturity group, breeding institution, country of origin, geographic region and pedigree

No.	Genotype name	Status	MG	Breeding institution	Country of origin	Geographic region	Pedigree
1	Progres	V	00^{-}	Instytut Hodowli i Aklimatyzacji Roślin – Państwowy Instytut Badawczy	Poland	EEA	IHAR 78-B
2	Agassiz	V	00	Minnesota AES	USA	USA	Simpson/N 71-148 (Simpson=Clay / Evans)
3	Evrika	V	00	РГП "Научно-Производственный Центр Земледелия и Растениеводства", Алматинская обл.	Kazakhstan	EEA	Beeson/Merit (Beeson=(Blackhawk/Harosoy)/Kent)
4	Gi 291/70-79	BL	00	Semundo Mais S.A.R.L.	France	EEA	Unknown
5	Kabott	V	00^{+}	Department of Agriculture,Central Experimental Farm, Otawa, Ontario	Canada	CAN	Selection from Manchurian line introduced to North America in 1933.
6	Ljuso	V	00	-	USA	USA	Unknown
7	Maple Arrow	V	00	Agriculture Canada, Research Station Otawa, Ontario	Canada	CAN	Harosoy 63 / Holmberg 840-7-3(PI 438 477)
8	Maple Presto	V	00^{-}	Agriculture Canada, Research Station Otawa, Ontario	Canada	CAN	(Amsoy/Portage)/Holmberg 840-7-3(PI 438 475)
9	Mini soja	PI	00	-	China	EXO	Unknown
10	Canatto	V	00	Eastern Cereal and Oilseed Research Centre, Agriculture and Agri-Food, Ottawa, Ontario	Canada	CAN	X655-2/Evans e3 (X655-2 small seed selection from DW-1-15-1/BD22115; DW-1-15-1 small seed selection from *G.soja* l M62-173; BD22115 – sister line of M.Presto)
11	PI 180 507	PI	00	-	China	EXO	Mandschurische Herkunft 238 / Mandschurische Herkunft 413
12	Korana	V	00	Agricultural Institute Osijek	Croatia	DOM	LP-57-95 / L-98RC
13	Olima	V	00	University of Giessen, Institute of Crop Production and Plant Breeding	Germany	EEA	Unknown
14	Krajina	V	00	Institute of Field and Vegetable Crops, Novi Sad	Serbia	DOM	Unknown
15	FS 2 78	BL	0	FS HiSoy, Growmark, Illinois	USA	USA	Unknown
16	Afrodita	V	0	Institute of Field and Vegetable Crops, Novi Sad	Serbia	DOM	S1346 / Hodgson
17	Apache	V	0	Eastern Cereal an Oilseed research Centre, Agriculture and Agri-food Canada, Otawa, Ontario	Canada	CAN	(Altona/Calland)/PI232997(Calland=(Blackhawk/Harosoy)/Kent)(Altona= Flambeau / 052-903 (= PI 194.654, Holmberg 753-1))
18	Atlas	V	0	Institute de cercetari pentru cereale di plante tehnice – Fundulea (NARDI)	Romania	EEA	(Portage/Merit)/(Tewels/K 125) (Merit= Blackhawk/Capital)
19	Aura	V	0	KWS	Germany	EEA	Unknown
20	Black Tokio	PI	0	Plant Gene Bank Yugoslavia	Japan	EXO	Unknown
21	Chandor	V	0	-	France	EEA	Unknown
22	Dawson	V	0	Minesota AES i USDA, USA	SAD	USA	Evans/M 63-217Y (=selekcija žutog hiluma iz Hodgson-a)
23	Lucija	V	0	Agricultural Institute Osijek	Croatia	DOM	(Bara / LR-66) / LR-66
24	F01- 484	V	0	Institute de cercetari pentru cereale di plante tehnice – Fundulea (NARDI)	Romania	EEA	(Danubian/Jilin)/(Ozzie/Victoria) (Ozzie=Wilkin/M 63-217Y)
25	Julijana	V	0	Agricultural Institute Osijek	Croatia	DOM	TISA / L-20245
26	Bačka	V	0	Institute of Field and Vegetable Crops, Novi Sad	Serbia	DOM	Unknown
27	Issik	V	0	-	Kazakhstan	EEA	Kyrgyz landrace
28	K-1	BL	0	-	Kazakhstan	EEA	Unknown
29	K 2 2	BL	0	-	Kazakhstan	EEA	Unknown
30	Kanadska 1	BL	0	-	Canada	CAN	Unknown
31	L 1128	BL	0	University of Illinois, Urbana, IL	USA	USA	Unknown
32	KWS Ilona	V	0	KWS	Germany	EEA	Unknown

Table S2. (continued)

No.	Genotype name	Status	MG	Breeding institution	Country of origin	Geographic region	Pedigree
33	Vita	V	0	Agricultural Institute Osijek	Croatia	DOM	L-492 / Drina
34	Lambert	V	0	Minesota AES	USA	USA	M75-274/M76-151 (M75-274=Evans/L70T-543G; M76-151=M70-271/Hodgson 78)
35	Lanka	V	0	Кировоградская государственная областная сельскохозяйственная опытная станция	Russia	EEA	(ВНИМК9186/Приморская 529)/(Кубанская 4958/Рекорд Северный)
36	OAC Eclipse	V	0	Department of Crop Science, University of Guelph, Guelph, Ontario	Canada	CAN	Maple Arrow/Williams (=Wayne/L57-0034 (=Clark/Adams))
37	L 7/88	BL	0	Селекционно-генетический институт Одесская областы Одесса	Russia	EEA	Unknown
38	PI 301	BL	0	-	Italy	EEA	Unknown
39	PRW 80	BL	0	-	Canada	CAN	Unknown
40	VNIMK 3895	BL	0	Всероссииски научно-исследовательский институт масличных культур имени В.С. Пустовойта, Краснодар	Russia	EEA	(ВНИИСК 7/Кормовая 1)/Комсомолна
41	Turska 1	V	0	-	Turkey	EEA	Unknown
42	Turska 2	V	0	-	Turkey	EEA	Unknown
43	Am 3	BL	0	-	USA	USA	Unknown
44	ZPS 015	V	0	Maize Research Institute Zemun Polje	Serbia	DOM	NBSG1 population
45	Kolubara	V	0	Institute of Field and Vegetable Crops, Novi Sad	Serbia	DOM	Unknown
46	Balkan	V	I	Institute of Field and Vegetable Crops, Novi Sad	Serbia	DOM	(Evans/Four)/S1346 (Evans=Merit/Harosoy; S1346=A55–4629–4/PI 257435; A55–4629=Roanoke/Hawkeye; A2506=NK 'S14–60'/ Asgrow 'A3501'.)
47	Chornaja	V	I	-	Bulgaria	EEA	Unknown
48	Danijela	V	I	-	Bulgaria	EEA	Unknown
49	Danubian	V	I	Institute de cercetari pentru cereale di plante tehnice–Fundulea (NARDI)	Romania	EEA	Peterson3100/(Tewels/K125)
50	Hodgson 78	V	I	Minnesota AES	USA	USA	Composite of 56 F_3 lines BC6 Merit/Hodgson(=Corsoy/M372 (=Lincoln/Richland)/PI 180.501(=Mandschurische Herkunft/USA54616)
51	Ardin	V	I	Rustica S.P.A.	Italy	EEA	Unknown
52	Krizia	V	I	Rustica S.P.A.	Italy	EEA	Unknown
53	OS 101	BL	I	Poljoprivredni institut, Osijek	Croatia	USA	Ika / L-125-08
54	Ravnica	V	I	Institute of Field and Vegetable Crops, Novi Sad	Serbia	DOM	Hodgson/ S1346 (S1346=A55–4629–4/PI 257435; A55–4629–4=Roanoke/Hawkeye)
55	Ika	V	I	Agricultural Institute Osijek	Croata	DOM	(Tisa/ L-1504) / L-OS-C-600
56	Shine	V	I	Rustica S.P.A.	Italy	EEA	Unknown
57	A 1937	V	I	Asgrow Seed Co., Kalamazoo, Michigan	USA	USA	Hodgson / L15 (=bc Wayne / Clark 63)
58	Laura	V	I	Maize Research Institute Zemun Polje	Serbia	DOM	Kunitz/Novka
59	NK 15 50	V	I	Northrup, King& Company Seed, Syngenta, Minesotta	USA	USA	Unknown
60	Parker	V	I	Minnesota AES	USA	USA	A79-136012/Dawson(A79-136012=B216/LandO'Lakes; B216=Corsoy/Wayne
61	Brock	V	I	Ridgetown College of Agricultural Technology and University of Guelph	Canada	CAN	B 152 / HW 8039 (=Weber/Pella)
62	A 3963	V	II^+	Asgrow Seed Co., Kalamazoo, Michigan	USA	CAN	Williams/Essex
63	Dekabig	V	II^-	Monsanto (DeKalb)	USA	USA	Unknown

Table S2. (continued)

No.	Genotype name	Status	MG	Breeding institution	Country of origin	Geographic region	Pedigree
64	FS BB	BL	II	FS HiSoy, Growmark, Illinois	USA	USA	Unknown
65	Gnome	V	II	Ohio ARDC and USDA	USA	USA	Williams/Ransom((=N55-5931/N55-3818)/D56-1185; N55-5931=D492491/Roanoke(=reselection from Chinese PI Nanking))
66	Harosoy	V	II	Department of Agriculture, Experimental Station Harrow, Ontario, Canada	Canada	CAN	Mandarin (Otawa) / A.K.(Harrow)
67	HS 302	V	II	FS HiSoy, Growmark, Illinois	USA	USA	Unknown
68	Kineska	PI	II	Plant Gene Bank Yugoslavia	China	EXO	Unknown
69	Nikko	V	II^-	Monsanto (Dekalb)	USA	USA	Unknown
70	Action	V	II	Rustica S.P.A.	Italy	EEA	Unknown
71	Beauty	V	II	Rustica S.P.A.	Italy	EEA	Unknown
72	J 4	BL	II	Jaques Seed Company, Prescott, Wisconsin	USA	USA	Unknown
73	Vertex	V	II	Ohio ARDC, Ohio State University	USA	USA	Conrad/Hayes (Conrad= (Williams/Essex)/(IVR/Calland); Hayes=Amcor/L24)
74	Volođa	V	II	Selsem, Delta Agrar	Serbia	DOM	Unknown
75	Vojvođanka	V	II	Institute of Field and Vegetable Crops, Novi Sad	Serbia	DOM	S1346/Hodgson
76	Lana	V	II	Maize Research Institute Zemun Polje	Serbia	DOM	Kunitz/Kador
77	Olga	V	II	Maize Research Institute Zemun Polje	Serbia	DOM	OS101/ZPS208 (=Hobbit/Platte)
78	Lidija	V	II	Maize Research Institute Zemun Polje	Serbia	DOM	(Sibley/A1937)/Kunitz (Sibley=(Evans/Steele)/Hodgson)
79	Nena	V	II	Maize Research Institute Zemun Polje	Serbia	DOM	OS101/Elf
80	Zen	V	II^-	Monsanto (Dekalb)	USA	USA	Unknown
81	Century	V	II	Purdue, AES, Indiana and USDA	USA	USA	Calland/Bonus(Calland=C1253(=Blackhawk/Harosoy)/Kent; Bonus=C1266R (=Harosoy/C1079) / C1253 (=Blackhawk/Harosoy))
82	Corsoy	V	II	Iowa AES and USRSL	USA	USA	Harosoy/Capital
83	KB 231	V	II	Kaltenberg Seed Farms, I.N.C.	USA	USA	Unknown
84	Elf	V	III	Illinois AES, Ohio ARDC i USDA	USA	USA	Williams/Ransom
85	Hobbit	V	III	Ohio, ARDC and USDA	USA	USA	Williams/Ransom
86	Kunitz	V	III	Illinois AES and USDA ARS	USA	USA	Williams 82/PI 157440 (Williams 82=BC6 Williams/Kingwa (Rps); PI 157440=Kum Du, S.Korea)
87	Sprite	V	III	ARS, USDA and Ohio ARDC	USA	USA	Williams/Ransom
88	PI 416 892	PI	III	-	Japan	EXO	Goyou Kuro Mame
89	Barc 11-X	PI	III^+	University of Illinois	USA*	EXO	Hobbit/L782206 (=L67L113/PI243541; PI 243541 je linija fasciata tipa stabla („Shakujo“, Japan))
90	Pixie	V	III^+	Ohio, ARDC and USDA ARS	USA	USA	Williams/Ransom

V – variety; BL – advanced breeding line; PI – exotic germplasm; MG – maturity group; AES – Agricultural Experimental Station; USDA – United States Department of Agriculture; ARS – Agricultural Research Service; ARDC – Agricultural Research and Development Center; * In Analysis of molecular variance classified into EXO group.
