# Supplementary material for: Depicting Soybean Diversity via Complementary Application of Three Marker Types
Source: Plants (Basel). 2025 Jan 12;14(2):201. doi: 10.3390/plants14020201 (PMC11768110; doi:10.3390/plants14020201)
Supplement: Supplementary file 1 [file plants-14-00201-s001.zip › Table S4.pdf]

Table S4. List of descriptors, categories, category-scores, and distribution of genotypes across descriptor categories

No.	Descriptor	Descriptor label	Categories	Category scores	Number of genotypes	Frequency (%)
1	Hypocotil color	HIPC	Absent	1	31	34
			Present	2	59	66
2	Habitus	H	Erect	1	27	30
			Erect to semi-erect	3	49	54
			Semi-erect	5	12	13
			Semi-erect to horizontal	7	2	22
3	Growth type	GT	Determinate	1	9	10
			Semideterminate	3	5	6
			Semideterminate to indeterminate	5	9	10
			Indeterminate	7	66	73
			Fasciata	9	1	1
4	Pubescence color	PUBC	Tawny	1	45	50
			Grey	2	45	50
5	Leaf blistering	LB	Absent or very weak	1	14	16
			Medium	3	54	60
			Strong	5	19	21
			Very strong	7	3	3
6	Shape of lateral leaflet	LLSh	Lanceolate	1	1	1
			Pointed ovate	5	47	52
			Rounded ovate	7	42	47
7	Size of lateral leaflet	LLS	Small	1	22	24
			Medium	3	33	37
			Large	5	35	39
8	Intensity of green color of leaf	ILC	Light	1	13	14
			Medium	3	47	52
			Dark	5	30	33
9	Flower color	FC	Violet	1	59	66
			White	2	31	34
10	Intensity of the brown color of the pod	IPC	Light	1	27	30
			Medium	3	33	37
			Dark	5	30	33
11	Seed shape	SSh	Spherical	1	16	18
			Spherical-flattened	3	41	46
			Elongated	5	19	21
			Elongated-flattened	7	14	16
12	Seed coat color	SCC	Yellow	1	72	80
			Yellow-green	3	10	11
			Green	5	4	4
			Black	7	3	3
			Brown	9	1	1
13	Seed coat luster	SL	Shiny	1	41	46
			Dull	2	49	54
14	Hilum color	HILC	Yellow	1	24	27
			Light brown	3	12	13
			Brown	5	15	17
			Dark brown	7	12	13
			Reddish	9	3	3
			Olive	11	4	4
			Grey	13	3	3
			Black	15	16	18
			Yellow to light brown	17	1	1
15	Color of hilum funicle	CHF	Same as testa	1	75	83
			Different to testa	2	15	17
