# Supplementary material for: Depicting Soybean Diversity via Complementary Application of Three Marker Types
Source: Plants (Basel). 2025 Jan 12;14(2):201. doi: 10.3390/plants14020201 (PMC11768110; doi:10.3390/plants14020201)
Supplement: Supplementary file 1 [file plants-14-00201-s001.zip › Table S5.pdf]

Table S5: Sequences of 21 SSR primers used in the analysis

No	Primer	Forward sequence*	Reverse sequence*	LG
1	Satt 147	CCATCCCTTCCTCCAAATAGAT	CTTCCACACCCTAGTTTAGTGACAA	D1a
2	Satt 172	AGCCTCCGGTATCACAG	CCTCCTTTCTCCCATTTT	D1b
3	Satt 009	CCAACTTGAAATTACTAGAGAAA	CTTACTAGCGTATTAACCCTT	N
4	Satt 194	GGGCCCAACTGATATTTAATTGTAA	GCGCTTTGTGTTCCGATTTTGAT	C1
5	Satt 225	AAAAATGTGTTAGAGCTTGTGTTGTTA	GCCACACTATTCCAGCCACTAC	A1
6	Satt 307	GCGCTGGCCTTTAGAAC	GCGTTGTAGGAAATTTGAGTAGTAAG	C2
7	Satt 308	GCGTTAAGGTTGGCAGGGTGGAAGTG	GCGCAGCTTTATACAAAAATCAACAA	M
8	Satt 329	GCGGGACGCAAAATTGGATTTAGT	GCGCCGAATAAAACGTGAGAACTG	A2
9	Satt 167	GATTTACGGGTACTTGGATTCAATA	AGCTACCCAATATGATACTCTACACAGT	K
10	Satt 173	TGCGCCATTTATTCTTCA	AAGCGAAATCACCTCCTCT	O
11	Satt 197	CACTGCTTTTTCCCCTCTCT	AAGATACCCCCAACATTATTTGTAA	B1
12	Satt 192	CACCGCTGATTAAGATTTTT	CGCTGAGTTGTTTTCATC	H
13	Satt 114	GGGTTATCCTCCCCAATA	ATATGGGATGATAAGGTGAAA	F
14	Satt 122	AACCAACTTGGGAATAGAC	GCTCTCTATCATTCACTAATCA	B2
15	Satt 045	TGGTTTCTACTTTCTATAATTATTT	ATGCCTCTCCCTCCT	E
16	Satt 406	GCGTGAGCATTTTTGTTT	TGACGGGTTTAATAGCAT	J
17	Satt 002	TGTGGGTAAAATAGATAAAAAT	TCATTTTGAATCGTTGAA	D2
18	Satt 191	CGCGATCATGTCTCTG	GGGAGTTGGTGTTTTCTTGTG	G
19	Satt 232	GCGGCGTGAATAGTATACGTTGAGA	GCGGACATAAATGCAATCACTTAAAAAG	L
20	Satt 127	CGCTTGTGAACCCTGCTAAA	CCATCCTCTGAAACCGTTATCT	I
21	Satt 228	TCATAACGTAAGAGATGGTAAAACT	CATTATAAGAAAACGTGCTAAAGAG	A2

* primer data obtained from http://soybase.org (accessed on June 2023.)
