# Supplementary figures and images for: Depicting Soybean Diversity via Complementary Application of Three Marker Types
Source: Plants (Basel). 2025 Jan 12;14(2):201. doi: 10.3390/plants14020201 (PMC11768110; doi:10.3390/plants14020201)

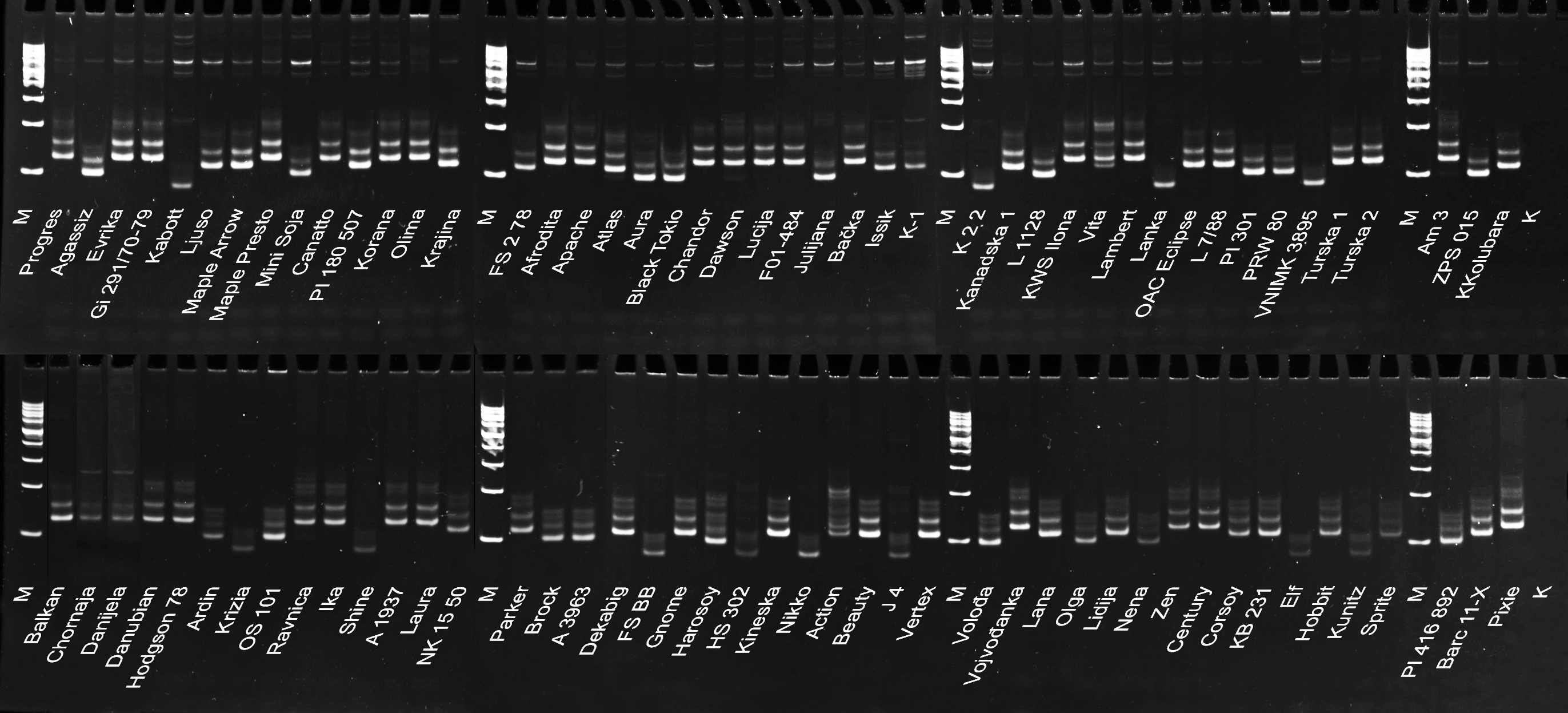

Supplement: Supplementary file 1 [file plants-14-00201-s001.zip › Figure S1.jpg]
